# A Thing of Beauty

**Published:** 1898-01

**Authors:** 


A Thing of Beauty.—To invest cod-liver oil with sentiment; to throw romance around its manufacture, might be looked upon as a triumph of genius, the subject is, within itself, so repulsive. It may be likened unto the sugar-coating of a bitter pill, or the whitewashing of an odoriferous reputation. But that is just what the indefatigable Parke, Davis & Co. have done. They have issued an illustrated brochure, beautifully—artistically illustrated, showing every step in the process of manufacturing their celebrated Lofotin cod-liver oil, from rigging out the schooner and starting before a spanking breeze to chase the festive cod in his habitat, all through the capture by hook and by seine of the old fellow; the dismemberment; the salting away; the return of the laden vessel over silvery seas and under the shade of shadowy mountains—a race for port; every detail, up to bottling and labeling the rich, pure amber-colored oil, so well known to the medical profession—the Lofotin. The little book is a gem,—a work of art worth preserving on one's center table for the pictures. But aside from that, the writer has invested the subject with an interest not thought to attach to so greasy a subject. A copy of the book can be had on application, free, on mentioning the Red-Back.

---

For a journal as ably conducted as has been the *New York Medical Journal* heretofore, I do think the editorial in the issue of December 25, ult., was a most puerile and weak one. It is on "The National Government and the Public Health," ostensibly; but it is a feeble argument in favor of enlarging the powers of the Marine Hospital Service—of giving the Secretary of the Treasury fuller powers in matters of—not public health—but of yellow fever; and the whole article reads as if the writer thought keeping yellow fever out was the only function to be performed by a bureau of public health. It is so clearly in the interest of the Marine Hospital Service as to lead one to suspect that it was written for the purpose, and is not a fair discussion of the question. It is in line with Cafferey's ridiculous bill. When the people are reminded that the South is indebted to that Marine Hospital Service for at least three outbreaks of yellow fever, they will not patiently bear further encroachments on State rights by this marine monster.

---

THE JOURNAL is in receipt of numerous letters relative to the tax on doctors. Among others recently received is one from a prominent physician in North Texas, who says that as soon as he had paid the tax—a county tax of $2.50, in addition to State tax of $5—he presented a just claim against the county for pauper practice, which he has been doing for years, free. The commissioners' court refused to pay it, alleging as a reason that it would make a precedent, and the county would hereafter have to pay for the pauper practice. Our correspondent adds: "Is it not a spectacle! A county taxing a man on his profession, and refusing to pay him for his services to the county." The JOURNAL suggests that all other physicians who are doing charity practice for the county, render bills and push their collection.

---

DR. T. J. TYNER, the well known oculist, of Austin, has removed to Washington City, where he will engage in the practice of his specialty. The JOURNAL extends its best wishes that the doctor may enjoy that full measure of success which his great merit and ability entitle him to. While we much regret losing him to Texas, we congratulate our Washington confreres on this valuable acquisition to their ranks. Dr. Tyner's improvement on cataract operation, a preliminary capsulotomy, an account of which he read at the ninth International Medical Congress, at Berlin, in 1892, has made him an international reputation as a skilled oculist. Treat him right, gentlemen; he is worthy of it.

Dr. J. Berrien Lindsley, the well-known physician and sanitarian of Nashville—long time President Tennessee State Board of Health, died at his home in that city Dec. 7, ult., aged 75 years.

